# Association Between Antenatal Exposure to Zika Virus and Anatomical and Neurodevelopmental Abnormalities in Children

**DOI:** 10.1001/jamanetworkopen.2020.9303

**Published:** 2020-07-07

**Authors:** Jessica S. Cranston, Sophia Finn Tiene, Karin Nielsen-Saines, Zilton Vasconcelos, Marcos V. Pone, Sheila Pone, Andrea Zin, Tania Saad Salles, Jose Paulo Pereira, Dulce Orofino, Patricia Brasil, Tara Kerin, Kristina Adachi, Fernanda Mendes Soares, Andrea Dunshee de Abranches, Ana Carolina C. da Costa, Maria Elisabeth Lopes Moreira

**Affiliations:** 1 Division of Infectious Diseases, Department of Pediatrics, David Geffen School of Medicine at University of California, Los Angeles; 2 Fundação Oswaldo Cruz, Rio de Janeiro, Brazil

## Abstract

**Importance:**

Zika virus (ZIKV) is a mosquito-borne flavivirus recognized as teratogenic since the 2015 to 2016 epidemic. Antenatal ZIKV exposure causes brain anomalies, yet the full spectrum has not been delineated.

**Objective:**

To characterize the clinical features of ZIKV infection at a pediatric referral center in Rio de Janeiro, Brazil, among children with antenatal ZIKV exposure.

**Design, Setting, and Participants:**

Retrospective cohort study conducted from May to July 2019 of a prospective cohort of 296 infants with antenatal ZIKV exposure followed up since December 2015 at a tertiary maternity-pediatric hospital.

**Exposures:**

Zika virus infection during pregnancy.

**Main Outcomes and Measures:**

Characterization of clinical features with anthropometric, neurologic, cardiologic, ophthalmologic, audiometric, and neuroimaging evaluations in infancy and neurodevelopmental assessments (Bayley Scales of Infant and Toddler Development, Third Edition) from 6 to 42 months of age, stratified by head circumference at birth (head circumference within the reference range, or normocephaly [NC] vs microcephaly [MC]).

**Results:**

Antenatal exposure to ZIKV was confirmed for 219 of 296 children (74.0%) referred to Instituto Fernandes Figueira with suspected ZIKV infection through positive maternal or neonatal polymerase chain reaction analysis or IgM serology results. Of these children, 110 (50.2%) were boys, ages ranged from 0 to 4 years, and 53 (24.2%) had congenital microcephaly. The anomalies observed in ZIKV-exposed children with MC or NC were failure to thrive (MC: 38 of 53 [71.7%]; NC: 73 of 143 [51.0%]), cardiac malformations (MC: 19 of 46 [41.3%]; NC: 20 of 100 [20.0%]), excess nuchal skin (MC: 16 of 22 [72.7%]; NC: 35 of 93 [37.6%]), auditory abnormalities (MC: 13 of 50 [26.0%]; NC: 14 of 141 [9.9%]), and eye abnormalities (MC: 42 of 53 [79.2%]; NC: 28 of 158 [17.7%]). Although they experienced fewer neurologic abnormalities than children born with MC, those with NC also had frequent neurologic abnormalities (109 of 160 [68.1%]), including hyperreflexia (36 of 136 [26.5%]), abnormal tone (53 of 137 [38.7%]), congenital neuromotor signs (39 of 93 [41.9%]), feeding difficulties (15 of 143 [10.5%]), and abnormal brain imaging results (44 of 150 [29.3%]). Among 112 children with NC with Bayley-III evaluations, 72 (64.3%) had average or above-average scores; 30 (26.8%) scored 1 SD below average in at least 1 domain; and 10 (8.9%) scored 2 SD below average in at least 1 domain. Among 112 children with NC, a smaller head circumference at birth was significantly associated with subsequent below-average cognitive scores (*U* = 499.5; *z* = −2.833; *P* = .004) and language scores (*U* = 235.5; *z* = −2.491; *P* = .01).

**Conclusions and Relevance:**

Children without MC who were exposed to ZIKV in utero had a high frequency of anatomical and neurodevelopmental abnormalities. The head circumference at birth for children with NC was associated with neurocognitive development. Recognition of the wide spectrum of clinical phenotypes is critical to ensure early referral to rehabilitative interventions.

## Key Points

**Question:**

What is the spectrum of clinical findings associated with antenatal Zika virus exposure, and does an association exist between head circumference at birth and neurodevelopmental outcomes among Zika virus–exposed children with normocephaly?

**Findings:**

This cohort study of 219 children found that antenatal Zika virus exposure was associated with a wide spectrum of clinical manifestations in normocephalic children beyond those previously described. The head circumference of normocephalic children was associated with neurodevelopmental outcomes.

**Meaning:**

Recognition of the variety of clinical phenotypes following antenatal Zika virus exposure, including evaluation of head circumference as a continuous variable, may help ensure early intervention, appropriate cross-disciplinary evaluation, and comprehensive therapeutic care.

## Teaser

This cohort study characterizes the clinical features associated with antenatal exposure to the Zika virus in children and assesses whether an association exists between head circumference at birth and neurodevelopmental outcomes.

## Introduction

Exposure to Zika virus (ZIKV) during pregnancy may lead to devastating brain damage in the infant, resulting in congenital ZIKV syndrome (CZS) and other clinical manifestations.^1,2,3,4,5,6,7,8,9,10,11,12,13,14,15,16,17^ Zika virus harms the developing brain by infecting human cortical neural progenitor cells and interfering with multiplication and migration of nervous system cells.^18,19,20^ A ZIKV epidemic in northeastern Brazil starting in May 2015 quickly spread to Rio de Janeiro between September 2015 and June 2016,^2,21,22,23,24,25,26^ with the virus identified by real-time reverse transcriptase–polymerase chain reaction (RT-PCR) in blood samples obtained from neonates with severe microcephaly and in amniotic fluid or tissue samples obtained from fetuses of women with rash during pregnancy.^14^ Instituto Fernandes Figueira (IFF) became a referral center for suspected cases of antenatal ZIKV infection in Rio de Janeiro.

Congenital ZIKV syndrome has been characterized by unique neurologic features,^2^ with other associated manifestations including seizures, developmental delay, auditory and visual deficits, dysphagia, intrauterine growth restriction, and fetal death.^27,28,29,30,31,32,33^ Infants antenatally exposed to ZIKV may not have findings of CZS because there is a wide spectrum of manifestations ranging from asymptomatic infection to microcephaly.^34^ Infants seemingly asymptomatic at birth may eventually develop abnormalities detected by brain imaging or in subsequent neurodevelopmental evaluations.^35,36^ Although microcephaly at birth is the hallmark of CZS, a wide spectrum of postnatal abnormalities have not yet been fully characterized.^28,35,36,37,38,39,40^ The primary objective of this study was to report the range of clinical manifestations in children with confirmed ZIKV antenatal exposure followed up at a large pediatric referral center in Rio de Janeiro.

## Methods

### Study Population

The study was conducted at IFF, Oswaldo Cruz Foundation (FIOCRUZ), in Rio de Janeiro, Brazil. Instituto Fernandes Figueira is a high-risk obstetric, perinatal, and pediatric referral center that follows a large number of cases of children with antenatal ZIKV exposure. The present study was a retrospective analysis focused on a prospective cohort of infants with ZIKV exposure with cases studied from December 2015 to July 2019.^27^ Infants with laboratory-confirmed ZIKV antenatal exposure were included. Pregnant women and infants included in the study received appropriate medical surveillance and supportive care because there is no specific treatment for ZIKV infection. Children were evaluated monthly for the first 6 months and then every 3 months. Follow-up assessments occurred depending on when parents were able to return to the clinic. The present report focuses on infants with microcephaly (MC) and those with normocephaly (NC), with clinical and neurodevelopmental data collected between the ages of 0 and 4 years. This cohort has 30% overlap with our prospective cohort of mother-infant pairs followed up over time for neurodevelopmental outcomes.^41^ The present cohort includes additional children born to mothers with asymptomatic infection or referred for abnormalities identified at birth. Data are reported according to the Strengthening the Reporting of Observational Studies in Epidemiology (STROBE) reporting guideline for cohort studies. Institutional review board approvals were obtained from IFF, FIOCRUZ and the University of California, Los Angeles. Written informed consent was obtained from parents or guardians in a manner consistent with the Declaration of Helsinki.^42^ No one received compensation or was offered any incentive for participating in this study.

### Laboratory Testing

Antenatal exposure to ZIKV was determined by RT-PCR testing of the mother and/or infant, as previously described, or infant IgM serologic testing (MacElisa, Centers for Disease Control and Prevention) at IFF.^27,43,44,45^ For some women who delivered at IFF, amniotic fluid and placental specimens were also tested by RT-PCR. Infants delivered at IFF had RT-PCR performed using urine, blood, or cerebrospinal fluid samples when available.^44^ Maternal and infant samples were also tested for HIV antibodies, cytomegalovirus IgM, parvovirus B19 IgM, Epstein-Barr virus serologic results, chikungunya PCR and IgM serologic results, dengue PCR results, venereal disease research laboratory results, and toxoplasma serologic results.^27^ All infants included in the present analysis had laboratory-confirmed ZIKV exposure during pregnancy, fulfilling at least 1 of the following criteria: (1) positive ZIKV PCR results during pregnancy from either serum, urine, placenta, or amniotic fluid samples; (2) positive IgM serologic results during pregnancy; (3) positive infant ZIKV PCR results at birth from serum, urine, or cerebrospinal fluid samples; or (4) positive infant IgM serologic results at birth.

### Infant Clinical Assessments

Anthropometric measures at birth (weight, length, and head circumference [HC]) were obtained for all live-born infants. Gestational age was measured by date of last menstrual period and by serial ultrasonography during pregnancy. The Ballard neonatal scale was used to assess gestational age at birth. Medical history and clinical assessments were conducted by pediatric specialists (ie, neonatologists [M.E.L.M.], neurologists [T.S.S.], cardiologists [D.O.], infectious disease specialists [M.V.P., S.P.], ophthalmologists [A.Z.], audiologists, geneticists, and physical therapists [F.M.S., A.D.A.]). Infants born with congenital anomalies were evaluated by a geneticist; the presence of a genetic illness was one of the exclusion criteria. Birth *z* scores were based on INTERGROWTH-21st Project data for gestational age and sex.^46^ Microcephaly was defined as a cephalic perimeter *z* score of less than −2 SD. Small-for-gestational-age (SGA) infants were defined as infants with body-weight *z* scores less than 1.28 at birth.^46^ Postnatal *z* scores were based on the Global Database on Child Growth and Malnutrition data from the World Health Organization (WHO).^47^ Failure to thrive was defined by 1 of the following criteria: (1) weight *z* score less than −1.89, (2) height *z* score less than −1.89, (3) weight-for-length *z* score of −1 or less, or (4) deceleration of weight-for-length *z* score of −1 or more (WHO anthropometric data used for weight-for-length variables at both time points). Nutritional and anthropometric assessments took place in earlier infancy to identify necessary interventions in developing infants. Anthropometric data at early infancy were evaluated as a potential factor associated with later development. Ophthalmologic outcomes were based on a complete eye examination with fundoscopy.^27,33,44,48^ Auditory outcomes were based on hearing assessments using brainstem evoked response audiometry.^27,41,48^ Neuromotor outcomes were based on physical examinations evaluating hypertonia or hypotonia, clonus, contractures or arthrogryposis, seizures, and continuous irritability (inconsolable crying).^27,41,48^ Cardiac outcomes were based on results of echocardiography performed by pediatric cardiologists (including D.O.) (eTable 1 in the Supplement).^40^ Blood tests were used to detect anemia and other hematologic abnormalities. The most recent assessment was included for all evaluations, with children ranging in age from 0 to 4 years.

### Neurodevelopmental Evaluation

The Bayley III Scales of Infant and Toddler Development, Third Edition [Bayley-III]^49^ were performed by trained neuropsychologists (F.M.S., A.D.A.) for children between 6 months and 3 years of age. The Bayley-III scales were used to assess 3 neurodevelopmental domains: cognitive, language, and motor functions.^50^ Below-average scores were defined as less than −1 SD to −2 SD below the mean, with scores less than 85 to 70 (at risk for developmental delay). Very below-average scores were defined as less than −2 SD below the mean, with scores less than 70 (developmental delay).

### Infant Imaging Studies

Brain imaging studies were offered to all infants, with screening transfontanelle ultrasonography (TFUS) routinely performed for ZIKV-exposed infants followed by further central nervous system imaging (computed tomography [CT] or magnetic resonance imaging [MRI]) when clinically indicated.^51^ Data on the abnormalities observed on neuroimaging are given in eTable 2 and eTable 3 in the Supplement.

### Statistical Analysis

Associations between clinical manifestations and Bayley-III scores were examined as 2-sided hypothesis at 80% power and 5% α levels. Bayley-III scores were examined as binomial values (abnormal or normal results) and continuous variables. Clinical manifestations were considered binomial (abnormal or normal results), with the exception of HC *z* scores, which were examined continuously. The Pearson χ^2^ test was used to investigate associations between clinical manifestations and Bayley-III scores when both were considered binomial variables. The Fisher exact test was used when appropriate. We used *t* tests to examine Bayley-III scores between abnormal and normal clinical groups, with Mann-Whitney tests used when appropriate. For associations between HC *z* scores and Bayley-III scores, correlation tests were used to determine *R* and *R^2^* values, and simple linear regression was used to determine *P* values. All analyses were performed with SPSS, version 25.0 (SPSS Inc).

## Results

**Figure 1.**
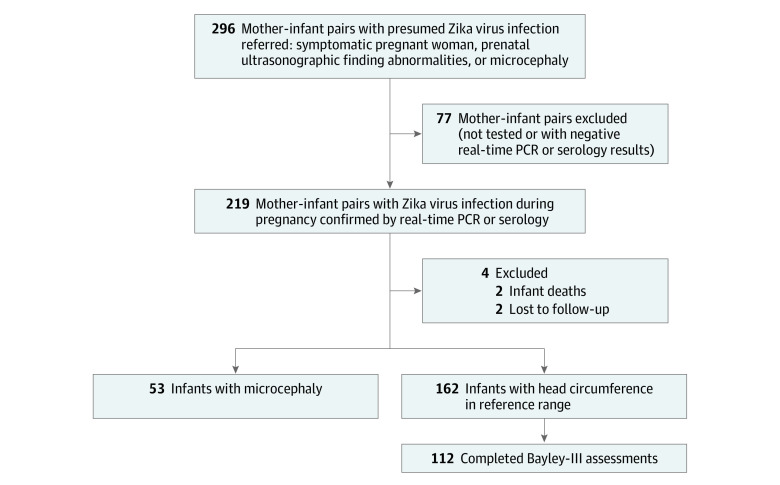
Flowchart of Included Participants PCR indicates polymerase chain reaction. ^a^Two infants died within 24 hours of birth, and 2 infants were lost to follow-up at birth; head circumference was not recorded for any of these 4 infants. Prebirth data of these infants were included in our study. Postbirth outcomes were not used owing to limited data.

Between September 2015 and June 2017, 296 children were referred to IFF for suspected antenatal ZIKV exposure (ie, 296 cases). Of these children, 150 (50.7%) were boys. Referrals were based on abnormal prenatal ultrasonographic findings suggestive of fetal ZIKV infection, maternal nonspecific viral symptoms, or laboratory assay results positive for ZIKV during pregnancy. The majority of women were referred because of positive maternal ZIKV PCR results. Most women were symptomatic because PCR tests were not otherwise readily available during the epidemic. Of 80 women examined at the IFF obstetrics clinic, 62 (77.5%) had abnormal ultrasonographic results. These included 48 of 66 infants (72.7%) with NC and 14 of 14 infants (100%) with MC. Antenatal exposure to ZIKV was confirmed in 219 cases (74.0%) through positive maternal or neonatal PCR or IgM serology results; 173 cases were confirmed by PCR of maternal serum or urine samples or placenta or amniotic fluid samples; 47 cases were confirmed by PCR of infant serum, urine, or cerebrospinal fluid samples; 48 cases had positive ZIKV IgM serologic test results, 4 in maternal specimens and 44 in infant specimens, for a total of 268 ZIKV-positive assay results from 219 mother-infant pairs; 110 children (50.2%) were boys. Birth data were available for 215 of 219 infants with laboratory-confirmed ZIKV exposure, including 2 deaths and 2 infants lost to follow-up shortly after birth. Fifty-three infants (24.7%) had congenital MC. Of 162 infants with NC, 112 (69.1%) completed Bayley-III evaluations (Figure 1).

Of 219 children, 198 (90.4%) had data on the timing of maternal infection in pregnancy: 72 (36.4%) were infected in the first trimester of pregnancy, 88 (44.4%) in the second trimester, and 38 (19.2%) in the third trimester. Among 53 children with MC, 42 had known timing of maternal infection during pregnancy; 34 (81.0%) were infected in the first trimester of pregnancy, 7 (16.7%) were infected in the second, and 1 (0.02%) was infected in the third. Nine additional patients were asymptomatic, and the trimester of infection was not ascertained; 2 additional patients did not have the information available in their medical records. There was a statistically significant association between maternal infection during the first trimester and MC (χ^2^ = 49.46; *P* < .001). For 198 children with maternal data available, the mean (SD) maternal age was 29.4 (6.3) years. Parity ranged from 0 to 6, with a mean (SD) of 1.9 (1.02). The number of live children per mother ranged from 0 to 5, with a median (SD) number of 1.6 (0.82).

Of 216 children with birth data available, 33 (15.3%) were born prematurely (<37 weeks’ gestation). The mean (SD) gestational age was 37.9 (3.3) weeks. For infants with NC, the mean (SD) HC *z* score at birth was 0.84 (1.24), the mean (SD) birth weight *z* score was 0.14 (1.09), and the mean (SD) birth height *z* score was 0.08 (1.32) (Table). For infants with MC, the mean (SD) HC *z* score at birth was −3.56 (0.88) (severe microcephaly), the mean (SD) birth weight *z* score was −1.30 (1.00), and the mean (SD) birth height *z* score was −1.09 (1.37). Of 160 infants with NC, 16 (10.0%) were SGA, and of 53 infants with MC, 30 (56.6%) were SGA. Antenatal exposure to ZIKV may cause fetal growth restriction; therefore, high rates of SGA were not surprising. Head circumference was not static; 17 of 162 children with NC at birth (10.5%) developed MC during follow-up. Conversely, MC resolved in 4 (7.5%) of 53 infants who were microcephalic at birth (proportional microcephaly). No mother-infant pairs in the cohort were diagnosed as having coinfections.

**Table.** Frequency of Abnormal Clinical and Laboratory Findings in Children With Antenatal ZIKV Exposure

Variable	Children with MC		Children with NC		All children	
	No./total No.	% (95% CI)	No./total No.	% (95% CI)	No./total No.	% (95% CI)
Death	4/52	7.7 (0.5 to 14.9)	2/160	1.3 (0 to 3.0)	8/214^a^	3.7 (1.2 to 6.3)
Premature	6/53	11.3 (2.8 to 19.9)	27/162	16.7 (10.9 to 22.4)	33/215	15.3 (10.5 to 20.2)
*z* Score at birth, mean						
Weight	−1.30	(−1.6 to −1.0)	0.14	(0.1 to 0.2)	−0.21	(−0.2 to −0.2)
Head circumference	−3.56	(−3.8 to −3.3)	0.84	(0.7 to 0.9)	−0.25	(−0.3 to −0.2)
Height	−1.09	(−1.5 to −0.7)	0.08	(−0.02 to 0.2)	−0.22	(−0.3 to −0.1)
Failure to thrive	38/53	71.7 (59.6 to 83.8)	73/143	51.0 (42.3 to 59.2)	111/196	56.6 (49.7 to 63.6)
Weight *z* score <−1.89 at follow-up	33/46	71.7 (58.7 to 84.8)	28/139	20.1 (13.5 to 26.8)	61/185	33.0 (26.2 to 39.8)
Height *z* score <−1.89 at follow-up	27/47	57.5 (43.3 to 71.6)	18/143	12.6 (7.2 to 18.0)	45/190	23.7 (18.0 to 29.4)
Weight-for-height *z* score <1 at follow-up	7/45	15.6 (4.97 to 26.2)	33/139	23.7 (16.7 to 30.8)	40/184	21.7 (16.2 to 27.2)
Deceleration of weight-for-height *z* score <1	4/36	11.1 (0.8 to 21.4)	37/131	28.2 (20.5 to 36.0)	41/167	24.6 (18.0 to 31.1)
Cardiac abnormality	19/46	41.3 (27.1 to 55.5)	20/100	20.0 (12.2 to 27.8)	39/146	26.7 (19.5 to 33.9)
Auditory abnormality	13/50	26.0 (13.8 to 38.2)	14/141	9.9 (5.0 to 14.9)	27/191	14.1 (9.4 to 18.8)
Ophthalmologic abnormality	42/53	79.2 (68.33 to 90.2)	28/158	17.7 (11.8 to 23.7)	70/211	33.2 (26.9 to 39.5)
Abnormal physical examination results	16/22	72.7 (54.1 to 91.3)	39/93	41.9 (31.9 to 52.0)	55/115	47.8 (41.1 to 54.5)
Excess skin on neck	16/22	72.7 (54.1 to 91.3)	35/93	37.6 (27.8 to 47.5)	51/115	44.3 (37.6 to 51.0)
Beak deformity of occipital bone	14/22	63.6 (43.5 to 83.7)	15/93	16.1 (8.7 to 23.6)	29/115	25.2 (19.4 to 31.0)
Neurologic abnormalities	53/53	100 (100 to 100)	109/160	68.1 (60.9 to 75.4)	162/213	76.1 (70.4 to 81.8)
Neuromotor abnormality	46/46	100 (100 to 100)	89/139	64.0 (56.1 to 72.0)	135/185	73.0 (67.0 to 79.0)
Hyperresponsive	39/46	84.8 (74.4 to 95.2)	45/137	32.9 (25.0 to 40.7)	84/183	45.9 (39.2 to 52.6)
Hyperreflexia	37/46	80.4 (69.0 to 91.9)	36/136	26.5 (19.1 to 33.9)	73/182	40.1 (33.5 to 46.7)
Hyperexcitability	32/46	69.6 (56.3 to 82.9)	21/137	15.3 (9.3 to 21.4)	53/183	29.0 (22.9 to 35.1)
Abnormal tone	45/46	97.8 (93.6 to 100)	53/137	38.7 (30.5 to 46.8)	98/183	53.6 (46.9 to 60.3)
Hypotonia	16/46	34.8 (21.0 to 48.6)	21/137	15.3 (9.3 to 21.4)	37/183	20.2 (14.8 to 25.6)
Hypertonia	10/46	21.7 (9.8 to 33.7)	35/137	25.6 (18.2 to 32.9)	45/183	24.6 (18.8 to 30.4)
Other congenital neuromotor signs	10/23	43.5 (23.2 to 63.7)	39/93	41.9 (31.9 to 52.0)	49/116	42.2 (35.6 to 48.8)
Fovea sign of the flexor regions	10/20	50.0 (28.1 to 72.0)	39/93	41.9 (31.9 to 52.0)	49/113	43.4 (36.7 to 50.1)
Arthrogryposis	5/23	21.7 (4.9 to 38.6)	2/93	2.2 (0 to 5.1)	7/116	6.0 (2.8 to 9.2)
Abnormal neurodevelopment	42/46	91.3 (83.2 to 99.5)	28/115	24.4 (16.5 to 32.2)	70/161	43.5 (36.8 to 50.2)
Abnormal feeding	16/47	34.0 (20.5 to 47.6)	15/143	10.5 (5.5 to 15.5)	31/190	16.3 (11.3 to 21.3)
Nystagmus	5/26	19.2 (4.08 to 34.4)	3/51	5.9 (0 to 12.3)	8/77	10.4 (6.3 to 14.5)
Seizures	21/46	46.7 (31.3 to 60.05)	5/137	3.7 (0.5 to 6.8)	26/183	14.2 (9.5 to 18.9)
Abnormal neuroimaging findings	51/53	96.2 (91.1 to 100)	44/150	29.3 (22.1 to 36.6)	95/203	46.8 (39.9 to 53.7)
Abnormal transfontanellar ultrasonographic findings	44/46	95.7 (89.8 to 100)	30/141	21.3 (14.5 to 28.0)	74/187	39.6 (33.0 to 46.2)
Abnormal brain CT findings	50/50	100 (100 to 100)	23/50	46.0 (32.2 to 59.8)	73/100	73.0 (64.3 to 81.7)
Abnormal brain MRI findings	23/23	100 (100 to 100)	16/43	37.2 (22.8 to 51.7)	39/66	59.1 (47.2 to 71.0)
Anemia	8/29	27.6 (11.3 to 43.8)	5/45	11.1 (1.9 to 20.3)	13/74	17.6 (12.5 to 22.7)
Neutropenia	5/29	17.2 (3.5 to 31.0)	7/45	15.6 (5.0 to 26.1)	12/74	16.2 (11.2 to 21.2)
Neutrophilia	16/30	53.3 (35.5 to 71.2)	14/45	31.1 (17.6 to 44.6)	30/75	40.0 (33.4 to 46.6)
Thrombocytosis	13/29	44.8 (26.7 to 62.9)	16/45	35.6 (21.6 to 49.5)	29/74	39.2 (32.6 to 45.8)
Thrombocytopenia	3/29	10.3 (0 to 21.4)	5/45	11.1 (1.9 to 20.3)	8/74	10.8 (6.6 to 15.0)

Abbreviations: CT, computed tomography; MC, microcephaly; MRI, magnetic resonance imaging; NC, normocephaly; ZIKV, Zika virus.

^a^ Two infants died without a measure of head circumference for a total of 8 pediatric deaths (4 infants with MC, 2 infants with NC, and 2 infants without head circumference results).

Of 213 ZIKV-exposed infants who underwent neurologic examinations, 162 (75.4%) had abnormal neurologic results. Of 160 infants with NC, 109 (68.1%) had abnormal neurologic findings. These included hyperreflexia (36 of 136 [26.5%]), abnormal tone (53 of 137 [38.7%]), and congenital neuromotor signs (39 of 93 [41.9%]), including fovea sign of the flexor regions (39 of 93 [41.9%]) and arthrogryposis (2 of 93 [2.2%]). All 53 children with MC (100%) had abnormal neuromotor findings. Neurologic abnormalities on physical examination were identified at birth for infants with MC and during follow-up for children with NC, typically at 3 months of age.

Of 203 children with neuroimaging studies performed, 95 (46.8%) had abnormal neuroimaging results. This included 51 of 53 children with MC (96.2%) and 44 of 150 (29.3%) children with NC. Children with abnormal TFUS results were referred for CT, MRI, or both. Two infants with MC with normal neuroimaging findings had normal TFUS results and did not undergo additional neuroimaging. Both were SGA and had proportional MC. The main neuroradiologic findings were brain calcifications identified on 47 of 188 (25.0%) TFUS scans, 22 of 66 (33.3%) MRI scans, and 54 of 100 (54.0%) CT scans. Other common findings were cerebral atrophy identified on MRI scans (16 [24.2%]) and CT scans (46 [46.0%]), ventriculomegaly identified on MRI scans (11 [16.7%]) and CT scans (40 [40.0%]), lissencephaly identified on MRI scans (14 [21.2%]) and CT scans (19 [19.0%]), and ventriculomegaly identified on TFUS scans (22 [11.7%]). Ventriculomegaly was defined based on clinical review by neuroradiologists. Of 50 children with NC, 23 (46.0%) had abnormal CT results, with calcifications being most prominent (14 [28.0%]). Of 43 children with NC, 16 (37.2%) had abnormal MRI findings, with calcifications (4 [9.3%]) and cerebral atrophy (4 [9.3%]) being most frequent.

**Figure 2.**
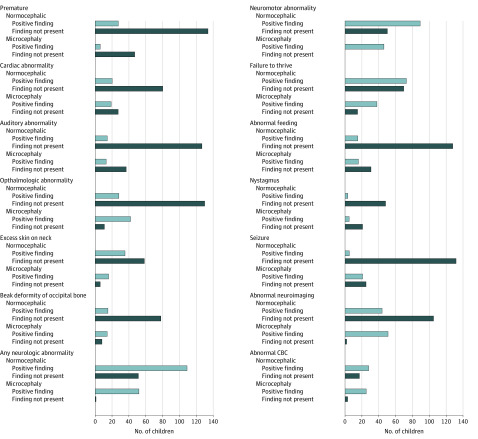
Frequency of Abnormal Findings in Zika Virus–Exposed Children Categorized by Head Circumference at Birth Bars depict the total number of children evaluated in each category stratified by head circumference. CBC represents complete blood count.

Nutritional assessments were performed for 143 children with NC between the ages of 0 and 16 months (mean [SD] age, 3 [4] months). Approximately half of the children (73 [51.0%]) had failure to thrive, with 15 children (10.5%) having abnormal feeding as defined by dysphagia, altered swallowing, and altered suction (Table). Among 73 infants with NC and failure to thrive, 15 (20.5%) developed secondary MC. Among children born with MC, 38 (71.7%) had failure to thrive, for a total of 111 children (56.6%) with failure to thrive in this cohort. In addition, 20 of 100 infants (20.0%) had cardiac abnormalities; 14 of 141 infants (9.9%) had abnormal hearing, 28 of 158 children (17.7%) had abnormal eye examinations, 35 of 93 children (37.6%) had excess nuchal skin, with 15 (16.1%) having beak deformity of the occipital bone; and 3 of 51 children (5.9%) had nystagmus (Table). Abnormal findings were present at higher frequencies among children with MC (Figure 2).

Of 77 infants with blood test results, 13 (16.2%) had anemia, 12 (16.2%) had neutropenia, 32 (41.6%) had neutrophilia, 29 (39.2%) had thrombocytosis, and 8 (10.8%) had thrombocytopenia in the first 6 months of life. Of 77 infants with blood tests, 45 (58.4%) were normocephalic.

**Figure 3.**
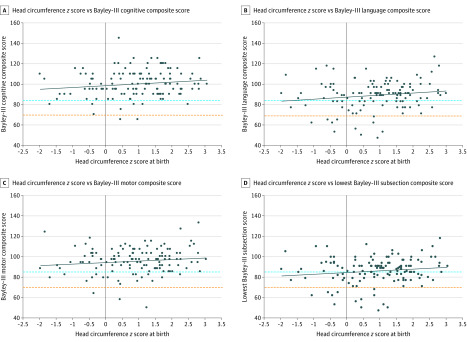
Individual Scores on the Bayley Scales of Infant and Toddler Development, Third Edition (Bayley-III), According to Head Circumference *z* Score at Birth of Children With Normocephaly Scores below 85 (blue dashed line) indicate risk of developmental delay; scores below 70 (orange dashed line) indicate risk of severe developmental delay. Each dot represents the most recent Bayley-III score of 1 of 112 children with normocephaly. Each solid line represents the line of best fit (correlation coefficient *R*^2^). Among children with normocephaly, a smaller head circumference (*z* score) at birth was significantly associated with “below-average” Bayley-III cognitive scores (*U* = 499.5; *z* = −2.833; *P* = .004) and language scores (*U* = 235.5; *z* = −2.491; *P =* .01).

Children with MC were developmentally unable to participate in Bayley-III assessments; therefore, none had Bayley-III results. Among 112 children with NC who underwent Bayley-III testing, 72 (64.3%; 95% CI, 55.4%-73.2%) had Bayley-III scores above or equal to 85 (≥−1 SD) for all 3 domains, 30 (26.8%; 95% CI, 18.6%-35.0%) had 1 or more Bayley-III scores between 84 and 70 (from −1 SD to −2 SDs), and 10 (8.9%; 95% CI, 3.7%-14.2%) had 1 or more scores below 70 (<−2 SDs), indicating developmental delay. The mean (SD) Bayley-III scores in the 3 domains for infants with NC were 99.91 (13.32) for cognitive, 89.12 (13.95) for language, and 95.43 (11.82) for motor. For children with NC and Bayley-III evaluations, a larger HC at birth (*z* score) was associated with improved overall scores (*U* = 0.014; *z* = −2.414; *P* = .01). Among children with NC, a smaller HC at birth (*z* score) was significantly associated with abnormal Bayley-III cognitive scores (*U* = 499.5; *z* = −2.833; *P* = .004) and language scores (*U* = 235.5; *z* = −2.491; *P* = .01) but not motor scores (*U* = 111.5; *z* = −0.974; *P* = .35) (Figure 3).

## Discussion

In utero ZIKV exposure is associated with a variety of serious congenital clinical manifestations, called CZS when specific clinical findings are identified.^2^ The present study sought to describe the wider spectrum of clinical manifestations in children with in utero exposure to the virus who were born in the aftermath of the Rio de Janeiro ZIKV epidemic. We focused on a cohort of 219 children with laboratory-confirmed antenatal ZIKV exposure, although there were 77 additional children with suspected exposure without laboratory confirmation. The children in the present cohort exhibited a wide variety of congenital abnormalities and a high frequency of neurodevelopmental delay noted during neuropsychological testing. However, this was a retrospective analysis of children with confirmed ZIKV exposure with available follow-up referred to IFF, a pediatric tertiary center. Therefore, we could not extrapolate incidence data because the children arrived from different locations and the mothers were not necessarily followed up from the time of acute ZIKV infection. This is distinct from a longitudinal prospective cohort of 216 children from both FIOCRUZ sites in Rio de Janeiro followed up since the time of maternal infection in utero.^27,41,48^ The present study reported on all children with positive ZIKV results followed up at the IFF, FIOCRUZ pediatric site and includes children born to women with symptomatic or asymptomatic infections. Because infants were often referred owing to abnormalities noted during pregnancy, at birth, or shortly thereafter, unsurprisingly, the cohort was a heavily symptomatic group of children.

Previous reports have described myriad poor clinical outcomes for infants with antenatal exposure to ZIKV, including brain, ophthalmologic, hearing, and motor abnormalities; severe MC; and other serious central nervous system findings.^2,27,33,38,41,44,48,51,52^ Severe outcomes in fetuses or newborns exposed to ZIKV during pregnancy have been identified, including intrauterine growth restriction, cerebral calcifications, abnormal arterial flow in the cerebral or umbilical arteries, global cerebral atrophy, MC, macular hypoplasia and scarring, and placental insufficiency.^27^ In addition, the presence of congenital cardiac defects has been described in infants with in utero ZIKV exposure.^40^ Many previous descriptive studies have focused on children with MC; we expanded this literature by evaluating HC as a continuous variable and also stratifying outcomes according to the presence or absence of MC.

We noted that the present cohort had a high frequency of abnormal outcomes among children with antenatal ZIKV exposure but who did not have MC at birth. Approximately 68% of these children had neurologic abnormalities on physical examination, 30% had abnormal neuroimaging results, and 57% had failure to thrive because of neurologic repercussions leading to poor feeding. These infants had initially been perceived as developing normally based on HC. The definition of MC as a cephalic perimeter *z* score of less than −2 as an arbitrary cutoff did not clearly correlate with neurodevelopmental outcomes. Specifically, among children with NC, the HC *z* score at birth directly correlated with neurodevelopmental outcomes. Although many children with NC were not born with an obvious congenital birth defect, neurodevelopmental abnormalities were found in 36% of the children who later underwent Bayley-III assessments. These results illustrate that antenatal ZIKV exposure may be associated with a wide clinical spectrum, with children exhibiting a variety of manifestations and outcomes. Because long-term adverse outcomes stemming from antenatal ZIKV exposure are not yet known, careful monitoring and evaluation of children with suspected exposure is essential for ensuring early detection of possible disabilities and referral to interventional services that may improve outcomes.

### Limitations

The limitations of this study include those associated with observational cohorts. One potential limitation is that not every child underwent every clinical assessment. In addition, not every child underwent each modality of neuroimaging. Neonates with concerning findings in utero or at birth were more likely to undergo further evaluation with postnatal neuroimaging. Another limitation is that clinicians were not blinded as to whether a child was exposed to ZIKV in utero. Blinding to ZIKV status was impossible because every child born in Brazil during the epidemic was at risk of ZIKV antenatal exposure. Furthermore, the neurodevelopmental program was in place mainly for ZIKV-exposed children. We believe that there was no ascertainment bias associated with Bayley-III assessments because a strict protocol was followed for all children. Another limitation is that there was no control population. Given the difficulty of diagnosing ZIKV infection retrospectively in asymptomatic women, it could not be ascertained that a simultaneous control group of children was never exposed to ZIKV. Zika exposure is extraordinarily difficult to determine if patients are not tested by PCR during acute infection. Determining whether infants were positive for ZIKV was also difficult because virus shedding may have been intermittent or may have ceased by the time of birth. The IgM responses may have been delayed and, when present, are only detected in the first 3 months of infection. Furthermore, ZIKV IgG cross-reacts with dengue antibodies and is not effective for diagnosis. In addition, the frequency of abnormal clinical findings in our population greatly exceeded that observed in general populations. The present ZIKV cohort was more symptomatic because of referral patterns.

## Conclusions

Antenatal ZIKV exposure was associated with a wide spectrum of clinical outcomes. Although MC has become the hallmark of CZS and is associated with severe central nervous system outcomes, our findings suggested that ZIKV-exposed infants without MC experienced similar congenital symptoms at high frequencies. The HC *z* scores at birth among children with NC were associated with neurocognitive development, indicating that HC should be evaluated as a continuous variable in assessing neurodevelopmental risk. Recognition of diverse infant clinical phenotypes following maternal ZIKV infection during pregnancy, beyond MC, by pediatric health care clinicians may help ensure early intervention, appropriate cross-disciplinary evaluation, and comprehensive therapeutic care.

## Notes

Supplement. **eTable 1.** Cardiologic Abnormalities Identified **eTable 2.** Abnormal Transfontanellar Ultrasonography Findings **eTable 3.** Abnormal CT or MRI Findings
