# Supplementary material for: Association Between Antenatal Exposure to Zika Virus and Anatomical and Neurodevelopmental Abnormalities in Children
Source: JAMA Netw Open. 2020 Jul 7;3(7):e209303. doi: 10.1001/jamanetworkopen.2020.9303 (PMC7341180; doi:10.1001/jamanetworkopen.2020.9303)
Supplement: Supplement. — eTable 1. Cardiologic Abnormalities Identified eTable 2. Abnormal Transfontanellar Ultrasonography Findings eTable 3. Abnormal CT or MRI Findings [file jamanetwopen-3-e209303-s001.pdf]

Supplementary Online Content

Cranston JS, Tiene SF, Nielsen-Saines K, et al. Association between antenatal exposure to Zika virus and anatomical and neurodevelopmental abnormalities in children. *JAMA Netw Open*. 2020;3(7): e209303. doi:10.1001/jamanetworkopen.2020.9303

eTable 1. Cardiologic Abnormalities Identified

eTable 2. Abnormal Transfontanellar Ultrasonography Findings

eTable 3. Abnormal CT or MRI Findings

This supplementary material has been provided by the authors to give readers additional information about their work.

© 2020 Cranston, JS et al. *JAMA Network Open*.

eTable 1. Cardiologic Abnormalities Identified

Patent foramen ovale
Bicuspid aortic valve
Hypertrophic cardiomyopathy
Mitral Regurgitation
Tricuspid Insufficiency
Aortic Coarctation
Pulmonary Stenosis
Atrial Septal Defect
Ventricular Septal Defect

© 2020 Cranston, JS et al. *JAMA Network Open*.

eTable 2. Abnormal Transfontanellar Ultrasonography Findings

Microcephaly
Hydrocephaly
Cerebral Abnormality
Calcification
Other Abnormality

© 2020 Cranston, JS et al. *JAMA Network Open*.

eTable 3. Abnormal CT or MRI Findings

Pachygyria
Lissencephaly
Calcification
Blake’s Cyst
Ventriculomegaly
Dandy Walker
Cerebral Atrophy
Hydranencephaly
Other Abnormality

© 2020 Cranston, JS et al. *JAMA Network Open*.
